# Iatrogenic Splenic Injury Following Colonoscopy: A Rare Complication

**DOI:** 10.7759/cureus.96629

**Published:** 2025-11-11

**Authors:** Mohamed Elobaid, Ahmed I Idriss, Ahmed Sayedahmed, Fagr S Sadig, Muez A Ahmed, Yousif I Idriss, Abdallah Yousif

**Affiliations:** 1 General Surgery, Cavan General Hospital, Cavan, IRL; 2 Internal Medicine, Faculty of Medicine, University of Khartoum, Khartoum, SDN; 3 Internal Medicine, Omdurman Islamic University, Omdurman, SDN; 4 Faculty of Medicine, Sudan University of Science and Technology, Omdurman, SDN; 5 Surgery, Faculty of Medicine, University of Khartoum, Khartoum, SDN

**Keywords:** abdominal pain, colonoscopy complication, conservative management, hemoperitoneum, splenic injury

## Abstract

A 66-year-old woman developed severe epigastric pain and vomiting shortly after an outpatient colonoscopy. Initial clinical assessment and plain radiographs were inconclusive, but contrast-enhanced CT of the abdomen demonstrated a perisplenic hematoma with intraperitoneal blood consistent with splenic injury. She was hemodynamically stable on presentation and required blood transfusion for acute normocytic anemia. Given the absence of active arterial bleeding or peritonitis, the multidisciplinary team elected conservative management: hospital admission to a high-dependency unit for close monitoring, intravenous fluids, analgesia, broad-spectrum antibiotics, and serial hemoglobin and vital-sign assessments. No surgical or endovascular intervention was necessary; the patient’s symptoms and laboratory indices improved, and she was discharged in stable condition with follow-up arranged. This case highlights splenic injury as a rare but potentially serious complication of colonoscopy that may present without classic signs of perforation. Early recognition, appropriate imaging, and individualized management, particularly conservative care in selected stable patients, can avoid unnecessary surgery and reduce morbidity.

## Introduction

Splenic injury following colonoscopy is a relatively rare but potentially life-threatening complication, first documented in 1974 by Wherry and Zehner [1]. The most common complications associated with colonoscopy include perforation (0.1%-2.67%) and hemorrhage (0.001%-0.72%), as cited by multiple reviews of post-colonoscopy outcomes [2-4].

While rare, splenic injury is a serious concern. Several other uncommon complications have been reported in the literature, including mesenteric tears, portal vein gas, pneumomediastinum, pneumothorax, retroperitoneal emphysema, retroperitoneal abscess, hernia incarceration, septicemia, diverticulitis, appendicitis, and colonic volvulus [2]. Although splenic injury is not consistently listed among these rare complications, it remains an underrecognized and potentially fatal event. Some cases may go undetected or be misattributed to routine post-procedural discomfort-raising concerns in light of the increasing global use of colonoscopy for screening and diagnosis of colorectal pathology.

A 2014 systematic review of the literature examined 103 cases of splenic injury following colonoscopy, dating back 40 years to the first reported case by Wherry and Zehner [1,5]. These cases were documented across 75 publications in the form of case reports and case series. However, the true incidence remains unknown due to likely underreporting and diagnostic ambiguity.

The review found that 71.6% of affected patients were women, with a mean age of 63 years (range: 29-90 years). Of the cases in which surgical history was noted, 50.8% had undergone previous abdominal surgery, and 6.9% had prior pelvic surgery. Interestingly, more than half of the colonoscopies were performed for surveillance purposes [5].

Given the morbidity, potential mortality, and extended hospitalization associated with splenic injuries, it is essential to improve awareness of this complication and ensure it is well documented in clinical practice.

## Case presentation

In June 2025, a 66-year-old female, known to have rheumatoid arthritis and hypothyroidism, with no previous history of abdominal surgeries, was referred for gastroscopy and colonoscopy due to right upper quadrant and chest pain radiating to her back. Her general practitioner initially suspected a cardiovascular cause, given her past medical history of myxomatous mitral valve with mild regurgitation and a previous episode of transient Wenckebach block during a hospital admission for varicose vein treatment. She was referred to a specialist cardiac center where cardiac causes were ruled out, and gastroscopy, abdominal ultrasound, and colonoscopy were recommended to evaluate possible gallstones, duodenal ulcer, or alternating bowel habits.

Her past medical history also included chronic back pain and bilateral total hip replacements. At the time of presentation, she was not taking any anticoagulants, corticosteroids, or other immunosuppressive medications for rheumatoid arthritis.

**Figure 1:**
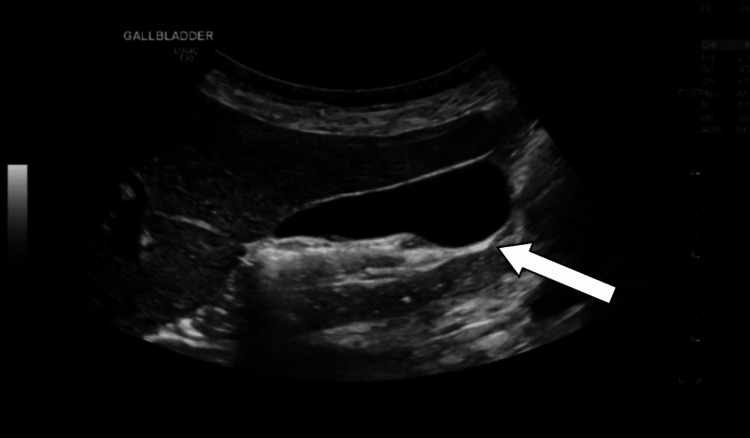
Ultrasound image obtained before colonoscopy showing the gallbladder (arrow) with normal appearance.

**Figure 2:**
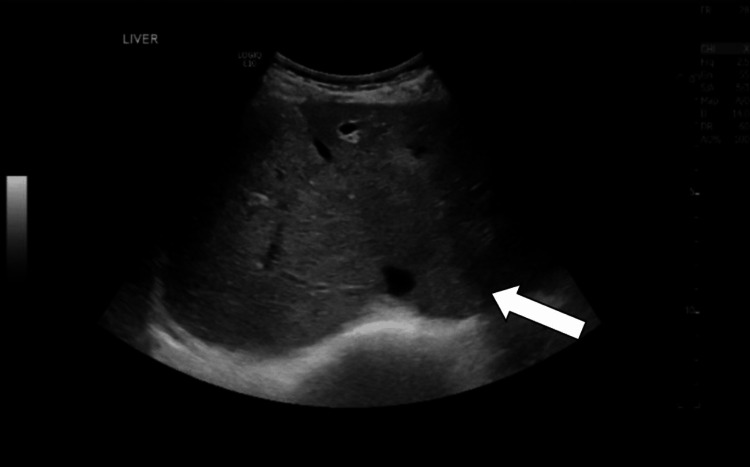
Ultrasound image obtained before colonoscopy showing the liver (arrow) with normal appearance.

**Figure 3:**
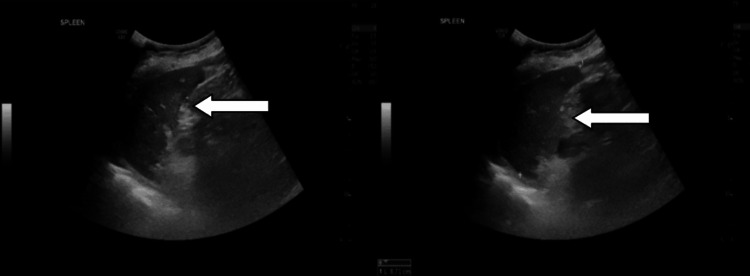
Ultrasound image obtained before colonoscopy showing the spleen (arrows) with normal appearance.

An abdominal ultrasound revealed a normal gallbladder and a common bile duct of normal caliber. The liver, spleen, and visualized portion of the pancreas appeared normal (Figures 1-3). The abdominal aorta was normal in caliber. Renal parenchyma was well-preserved bilaterally, with no evidence of renal calculi or hydronephrosis. Multiple bilateral renal cysts were noted, measuring up to 4.7 cm, but without concerning features.

Both esophagogastroduodenoscopy (OGD) and colonoscopy were performed by an experienced gastroenterology consultant under conscious sedation (midazolam 4 mg and fentanyl 100 mcg). Gastroscopy revealed a hiatus hernia and mild nodular antral and body gastritis, with a normal-appearing duodenum. Histology showed normal duodenal mucosa in the D2 biopsy, and a focus of intestinal metaplasia in the antral biopsy, but no evidence of atrophy, dysplasia, or malignancy. Helicobacter pylori testing was negative.

The colonoscopy report described a long, loopy, spastic, tortuous colon, requiring multiple positional adjustments to undo recurrent loops. The examination was otherwise normal up to the terminal ileum.

**Figure 4:**
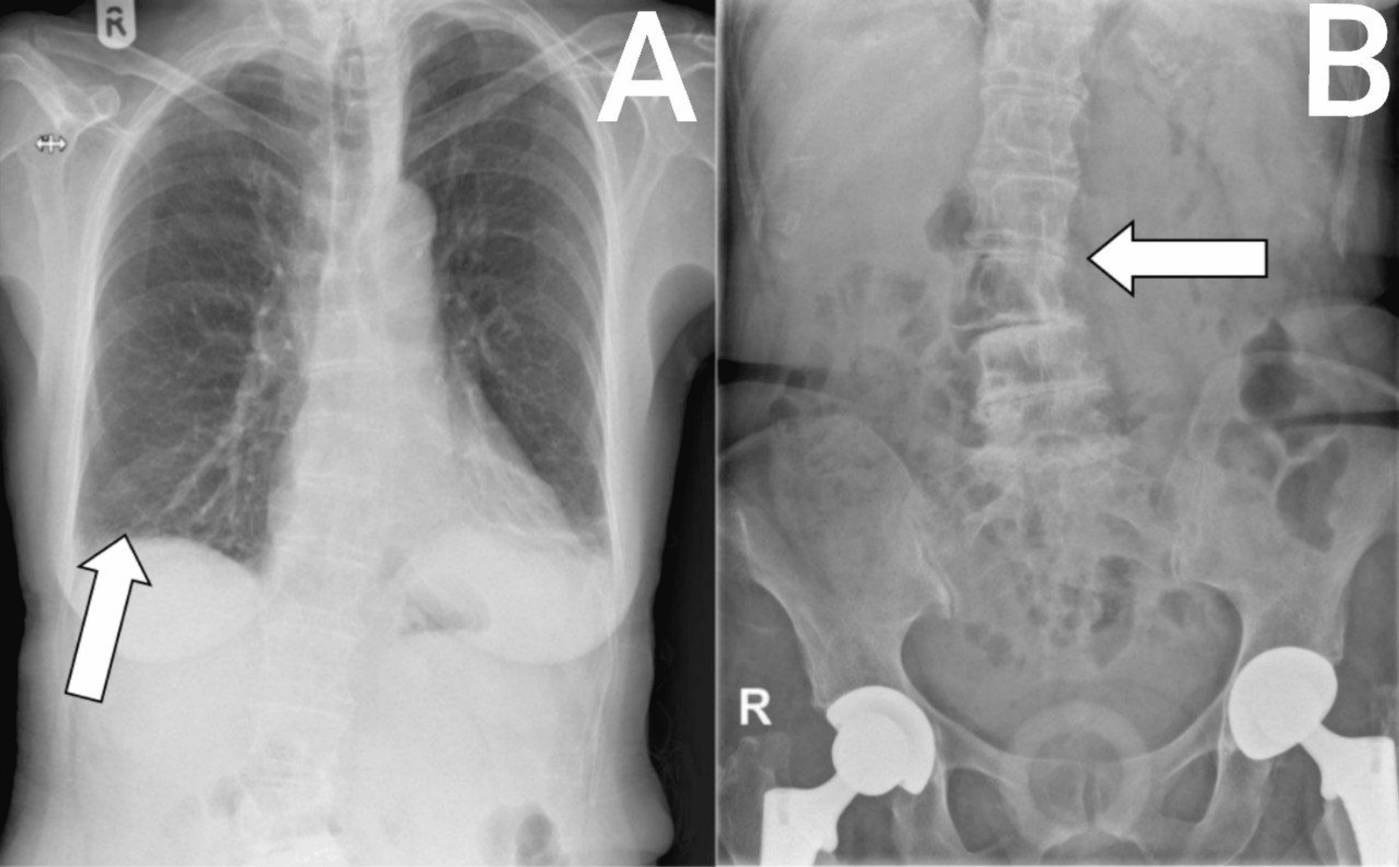
(A) Bilateral atelectasis is noted in lower zones (arrow in A) with no acute pulmonary infiltrate or pleural effusion and (B) the bowel gas pattern is unremarkable with no evidence of obstruction or perforation and scoliosis of the lumbar spine convex to the right is noted (arrow in B).

During recovery, the patient complained of severe epigastric pain (8/10) radiating to the left upper quadrant and left arm, associated with weakness. Her vital signs were initially within normal range. On examination, the abdomen was soft and non-tender, with no guarding, rigidity, or organomegaly, and bowel sounds were present. Concern for perforation or bleeding - the most common complications - led to an urgent erect chest and abdominal X-ray. The chest X-ray demonstrated bilateral basal atelectasis, with no acute infiltrate or effusion. The abdominal X-ray showed a normal bowel gas pattern, with no obstruction or perforation. Lumbar spine scoliosis convex to the right was also noted (Figure 4). Electrocardiogram (ECG) showed sinus rhythm with a prolonged PR interval (216 ms) and no evidence of ischemia.

Analgesics were prescribed; however, the epigastric pain persisted. Her pre-admission vital signs were blood pressure 150/80 mmHg, heart rate (HR) 58 bpm, oxygen saturation 100%, and temperature 36.3 °C. Intra-procedure vitals were stable, with systolic BP 140-150 mmHg, diastolic 70-95 mmHg, and HR 70-90 bpm. Post-procedure, her BP dropped to 103/57 mmHg, HR 70 bpm, respiratory rate (RR) 14 breaths per minute, with oxygen saturation 100% on 3 L/minute O₂. Over the next 24 hours, her BP further declined to 95/59 mmHg despite receiving 1 L of Hartmann’s solution (Table 1).

**Table 1:** Laboratory investigations following colonoscopy and at 24 hours post-colonoscopy. WCC, white cell count; HB, hemoglobin; RCC, red cell count; HCT, hematocrit; MCV, mean corpuscular volume; RDW, red cell distribution width; ESR, erythrocyte sedimentation rate; CEA, carcinoembryonic antigen; ALT, alanine aminotransferase; AST, aspartate aminotransferase; ALP, alkaline phosphatase; GGT, gamma-glutamyl transferase; Na, sodium; K, potassium; Ca, calcium

Investigation	24 hours post-colonoscopy	Following colonoscopy	Reference range
WCC	11.3 x 10^9^/L	8.5 x 10^9^/L	4.0-10.0
HB	11.2 g/dL	10.8 g/dL	12.0-15.0
Platelet count	235 x 10^9^/L	216 x 10^9^/L	150-410
RCC	3.43 x 10^12^/L	3.28 x 10^12^/L	3.8-4.8
HCT	32.1%	30.2%	36-46
MCV	93.6 fL	92.1 fL	83-101
RDW	12.7%	12.3%	11.6-14.0
Neutrophils	8.6 x 10^9^/L	6.8 x 10^9^/L	2.0-7.0
Lymphocytes	1.6 x 10^9^/L	0.9 x 10^9^/L	1.0-3.0
Monocytes	0.9 x 10^9^/L	0.5 x 10^9^/L	0.2-1.0
Eosinophils	0.2 x 10^9^/L	0.1 x 10^9^/L	0.02-0.5
Basophils	0.1 x 10^9^/L	0.1 x 10^9^/L	0.02-0.1
Amylase	37 U/L	-	-
C-reactive protein	<1 mg/L	6 mg/L	0-5.0
ESR	6 mm/1 hour	-	-
Carcinoembryonic antigen	3.70 ng/mL	-	-
Sodium	138 mmol/L	138 mmol/L	136-145
Potassium	3.7 mmol/L	4.3 mmol/L	3.5-5.1
Urea	6.6 mmol/L	3.2 mmol/L	3.5-7.2
Creatinine	78 umol/L	65 umol/L	49-90
Total protein	64 g/L	63 g/L	62-81
Albumin	39 g/L	37 g/L	32-46
Globulin	25 g/L	26 g/L	18-36
Bilirubin	10 umol/L	16 umol/L	3.4-20.5
ALT	20 U/L	-	-
AST	Specimen hemolyzed	Specimen hemolyzed	-
Alk Phos	42 U/L	42 U/L	46-122
Gamma GT	17 U/L	16 U/L	5-37
Calcium	2.13 mmol/L	2.09 mmol/L	2.10-2.55
Lactate	1.40 mmol/L	1.20 mmol/L	0.50-2.0
Random glucose	5.9 mmol/L	-	-

**Figure 5:**
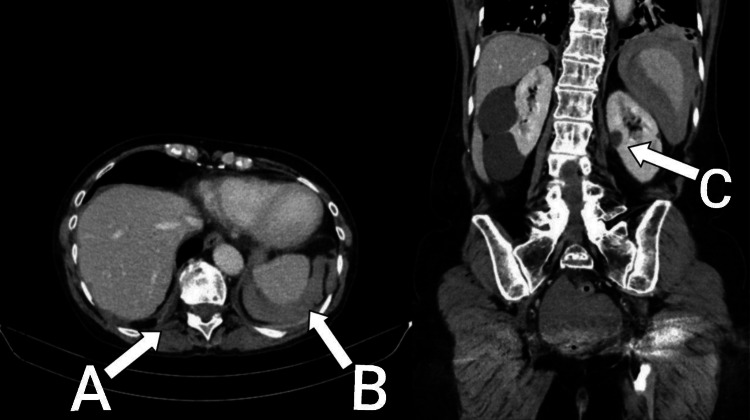
CT of the abdomen. Moderate-volume free fluid is present within the pelvis and upper abdomen, surrounding the liver and spleen. Around the liver, the fluid has a mean attenuation of approximately 55 Hounsfield units, suggesting complex rather than simple fluid (arrow A). The majority of high-attenuation free fluid, which could represent blood, surrounds the spleen in the left upper quadrant (arrow B), raising the possibility of hemoperitoneum secondary to splenic rupture. There is no evidence of free air beneath the right or left hemidiaphragm. Bilateral renal cysts are noted (arrow C), with no suspicious renal mass or hydronephrosis. Bilateral hip prostheses degrade image quality in the pelvis. There is no evidence of small or large bowel obstruction.

A CT of the abdomen revealed high-attenuation free fluid, predominantly surrounding the spleen in the left upper quadrant, consistent with hemoperitoneum secondary to splenic rupture (Figure 5).

Upon confirmation of the diagnosis, the patient was admitted to the high-dependency unit for close observation and hemodynamic monitoring. Conservative management included intravenous fluids, blood transfusions, and broad-spectrum antibiotics. No surgical intervention was required during her hospital stay, which lasted approximately seven days.

The patient’s condition gradually improved, and she was discharged in stable condition. At follow-up, she remained asymptomatic with complete recovery and no recurrence of symptoms.

## Discussion

Splenic injury following colonoscopy is a rare but potentially life-threatening complication, first reported by Wherry and Zehner in 1974 [1]. While colonoscopy is generally considered a safe procedure, the overall incidence of splenic injury remains extremely low, with only a few hundred cases reported in the literature [2,3]. The true incidence may be underestimated due to underreporting or misdiagnosis, as the symptoms may mimic more common post-procedural complications such as perforation or bleeding [4,5].

The mechanism of injury is not completely understood, but it is most commonly attributed to excessive traction on the splenocolic ligament, direct trauma to the splenic capsule, or torque applied during manipulation of the endoscope, particularly at the splenic flexure [6]. Other procedural factors - such as looping of the colonoscope, external abdominal pressure, and the use of the *hooking* or *slide-by* technique - may increase the risk of capsular tears or subcapsular hematomas [7]. In our patient, the colonoscopy report described a long, tortuous colon requiring multiple positional changes and loop reduction, which may have contributed to splenic stress and subsequent injury.

Patient-related risk factors reported in previous studies include female gender, prior abdominal or pelvic surgery, inflammatory bowel disease, anticoagulant use, and splenomegaly [8]. Although our patient did not have these predisposing factors, her presentation was consistent with previous reports describing left upper quadrant or referred shoulder pain (Kehr’s sign) within 24 hours post-procedure [9,10].

CT of the abdomen with contrast remains the diagnostic modality of choice, allowing identification of hematomas, capsular lacerations, and hemoperitoneum [9]. In this case, CT revealed a perisplenic hematoma and hemoperitoneum, confirming the diagnosis. The management of splenic injury depends on hemodynamic stability and injury severity. According to the American Association for the Surgery of Trauma (AAST) grading system, stable patients with low-grade injuries (Grades I-II) can be managed conservatively with observation, fluids, antibiotics, and transfusion if needed, while unstable or high-grade cases require surgical intervention [11]. Embolization may be considered in select cases with ongoing bleeding [12].

Our patient was successfully managed conservatively with blood transfusions, antibiotics, and close monitoring. This aligns with current recommendations emphasizing conservative management in stable patients. Clinicians should maintain a high index of suspicion for splenic injury when evaluating post-colonoscopy abdominal pain, particularly when accompanied by unexplained anemia or hemodynamic instability, as early recognition can prevent unnecessary morbidity or surgical intervention.

## Conclusions

Splenic injury remains an underrecognized and underreported complication of colonoscopy, requiring a high index of suspicion for early diagnosis and effective management. Although the association and identified risk factors between splenic injury and colonoscopy remain weak, this is mainly due to the very small number of reported cases. It is important to consider this complication in the setting of difficult colonoscopies, especially in patients with a history of abdominal surgery or those receiving anticoagulation therapy. Splenic injury should also be included in the differential diagnosis when evaluating unexplained epigastric pain or discomfort accompanied by hemodynamic instability and a fall in hemoglobin levels. With the increasing number of colonoscopies performed worldwide for both surveillance and diagnostic purposes, awareness of this rare but serious complication is essential, particularly for patients presenting within the first 24 hours post-procedure.
